# Author index

**DOI:** 10.1054/bjoc.2001.1930

**Published:** 2001


					AB01 Collaborators 2 (CT6)
Abdullah SY 71 (P148)
Aboagye EO 14 (2.1), 89 (P222)
Abounder R 83 (P198)
Abraham J 51 (P70), 104
(P276), 104 (P277)
Abraham JM 68 (P138)
Acres RB 25 (7.6)
Adams EJ 54 (P81)
Adams J 74 (P162)
Adams-Jones D 64 (P121)
Adeyemo D 42 (P39)
Adlard JW 63 (P119)
Agarwal R 26 (8.2)
A'Hern R 69 (P140), 69 (P141)
Ahmad T 47 (P58)
Aird RE 101 (P266)
Airley R 35 (P11)
Alexander FE 107 (P291)
Al-Husaini H 60 (P107)
Ali S 45 (P48), 45 (P49)
Allan B 92 (P233)
Allan JM 13 (1.7)
Allen-Mersh TG 43 (P40), 43
(P41)
Allibone R 108 (P293)
Al-Mazrouei M 105 (P282)
Al-Othman SF 60 (P107)
Alvarez-Vallina L 24 (7.4)
Aly A 41 (P32)
Ameyaw MM 41 (P35)
Amin S 68 (P136)
Amjadi P 43 (P40)
Anderson A 20 (5.2), 21 (5.4)
Anderson J 31 (10.3)
Anderson NG 45 (P51), 47
(P58)
Anderson T 21 (5.6)
Anlezark GM 98 (P255)
Ansell W 88 (P217),
103 (P274)
Anthony C 17 (3.7)
Anthony-Pillau R 44 (P44)
Appleton K 18 (4.1)
Araia R 43 (P41)
Aram H 50 (P66)
Archer VR 57 (P92)
Ardeshna KM 2 (CT7)
Armand J-P 94 (P239)
Arun C 33 (P1), 33 (P2), 33
(P3)
Asher-Dean R 80 (P185)
Ashley S 70 (P146)
Askill C 104 (P277)
Atkins H 81 (P188)
Atkinson A 26 (8.3)
Atkinson RJ 57 (P93)
Attenoos R 72 (P151)
Aveyard JS 80 (P184)
Azim-Araghi A 102 (P271)
Babb P 68 (P137)
Baker SD 94 (P238)
Balaji V 108 (P293)
Baldwin GS 41 (P32)
Balkwill FR 19 (4.6)
Baluch C 35 (P12)
Bandhu S 49 (P65)
Banks RE 13 (1.8)
Barber J 52 (P77)
Barbui T 61 (P108)
Bardos JI 109 (P296)
Barlow CS 60 (P106)
Barnes D 79 (P181)
Barraclough BR 55 (P85)
Barrett SV 75 (P165)
Barrett-Lee P 20 (5.3)
Barrio J 9 (S26)
Barrow D 99 (P259), 99 (P260)
Bartlett JMS 74 (P159), 88
(P218)
Barton DPJ 26 (8.1)
Bateman AC 12 (1.2)
Bates N 26 (8.1)
Bates T 21 (5.4)
Bathers S 50 (P67)
Baylin SB 4 (S7)
Beale P 57 (P93)
Beardsmore DM 12 (1.3)
Begent RHJ 98 (P256), 99
(P257)
Bell S 73 (P158)
Bell SM 18 (4.3), 41 (P33)
Bener A 105 (P281), 105 (P282)
Bennett S 27 (8.6)
Bennouna J 94 (P239)
Benson R 65 (P124)
Bentzen SM 68 (P135)
Beresford M 69 (P142)
Bergin O 51 (P71)
Berry PA 105 (P283)
Betts CD 53 (P80)
Bhatia J 98 (P256), 99 (P257)
Bibby MC 34 (P8), 92 (P231),
92 (P232)
Bicknell R 15 (2.7)
Bidmead M 15 (3.1)
Bigio IJ 27 (8.5)
Binley K 98 (P254)
Birch JM 107 (P290), 107
(P291)
Bissett D 21 (5.4)
Blair ME 84 (P200)
Blanchot G 94 (P239)
Blenkiron C 73 (P156), 73
(P157)
Bliss J 20 (5.3)
BNLI 2 (CT7)
Boddy AV 93 (P235)
Bodmer WF 39 (P25)
Bogle SE 72 (P154)
Bogush E 49 (P64)
Bogush T 49 (P64)
Bond SJ 68 (P135)
Bonifer C 40 (P28)
Bonvini P 91 (P228)
Bosu NK 49 (P65)
Boulos PB 89 (P221)
Boultbee J 26 (8.2)
Bowden S 61 (P111)
Bower M 61 (P109)
Bown SG 27 (8.5)
Bownes P 89 (P220)
Boxall J 51 (P73)
Boyd M 24 (7.1), 24 (7.2)
Boyd MT 81 (P189)
Boyle P 8 (S22)
Bozcuk H 58 (P98)
Bradley C 50 (P66)
Bradley CJ 72 (P154)
Bradshaw TD 103 (P272)
Brady ME 88 (P216),
93 (P234)
Bragg CM 104 (P279)
Brammer C 103 (P275)
Brammer CV 63 (P119)
Bransfield K 78 (P178)
Branston L 64 (P121), 69
(P139)
Branston LK 68 (P138)
Brewster A 104 (P276), 104
(P277)
Brewster AE 43 (P43), 64
(P121)
Briggs C 53 (P80)
Briggs GM 27 (8.5)
Brimmell M 23 (6.7)
Brison DR 27 (8.7)
Brock A 68 (P137)
Bromley M 35 (P11)
Brooks AC 34 (P5)
Brown BL 100 (P262)
Brown G 17 (3.7)
Brown J 67 (P133)
Brown K 61 (P110)
Brown KW 30 (10.1)
Brown M 3 (S3)
Brown NS 15 (2.7)
Brown R 18 (4.1), 18 (4.2), 25
(7.5), 75 (P165), 87 (P213), 89
(P221), 99 (P258)
Brown RSD 62 (P113), 68
(P137)
Browne HL 103 (P272)
Brownhill S 106 (P284)
Brunton VG 22 (6.2)
Bubnovskaya L 91 (P227)
Bugat R 94 (P240)
Bulusu VR 38 (P23)
Buluwela L 45 (P48), 45 (P49)
Bundred NJ 47 (P58)
Burchill S 88 (P215)
Burchill SA 31 (10.5), 31
(10.6), 32 (10.7), 105 (P283),
106 (P284), 106 (P285)
Burcombe RJ 26 (8.4), 50 (P68),
50 (P69)
Burgon K 53 (P79)
Burke B 15 (2.6), 35 (P12)
Burke MD 78 (P175)
Burkitt M 54 (P83)
Burnet NG 38 (P23)
Burns D 56 (P88)
Burns JL 39 (P27)
Burtles S 25 (7.7)
Burton K 38 (P23)
Bustin S 85 (P203)
Butler PC 78 (P175)
Cadet J 38 (P22)
Cahill D 16 (3.2)
Cairns D 84 (P201)
Cairns DP 107 (P290)
Calvert AH 81 (P188)
Cameron D 20 (5.2), 50 (P67)
Cameron DA 28 (9.4)
Campbell F 55 (P86)
Campbell L 99 (P260)
Campbell MJ 19 (4.4), 46 (P54)
Camplejohn R 79 (P181)
Camplejohn RS 82 (P194)
Canney P 50 (P67)
Cannings R 64 (P121)
Carey FC 12 (1.4), 13 (1.5)
Carli M 105 (P280)
Carlin S 24 (7.1), 24 (7.2)
Carmichael J 2 (CT6), 21 (5.4),
50 (P67), 96 (P248)
Carpenter R 85 (P203)
Carragher NO 22 (6.2), 82
(P192)
Carter M 108 (P293)
Cartwright RA 13 (1.7)
Case MC 60 (P105)
Caslin A 72 (P151)
Cassidy J 41 (P35)
Causevic M 12 (1.4)
Cawkwell L 90 (P225)
Cemazar M 98 (P253)
Chadderton N 14 (2.3)
Chalkidou K 88 (P216)
Chalmers A 36 (P16)
Author index to Volume 85
Authors' names are indexed by both page number and the abstract number in parentheses
110
British Journal of Cancer (2001) 85(Suppl 1), 110-116
(c) 2001 Cancer Research Campaign
doi: 10.1054/ bjoc.2001.1930, available online at http://www.idealibrary.com on
Chan KC 47 (P58)
Chan S 21 (5.4)
Chang J 61 (P108)
Chaplin DJ 48 (P61)
Chapman EJ 80 (P184)
Charnock FMC 26 (8.1)
Chaturvedi A 21 (5.4), 90
(P225)
Chaudhary SR 101 (P265)
Checkley D 34 (P7)
Chester JD 24 (7.4), 72 (P154)
Chester KA 98 (P256), 99
(P257)
Chetty U 21 (5.6)
Chiloeches A 5 (S11)
Chilton S 59 (P102)
Chinegwundoh F 88 (P217)
Ching HY 72 (P153)
Chinje EC 36 (P14)
Chong H 24 (7.4)
Christensen SB 7 (S18)
Chung P 65 (P126), 70 (P143)
Clackson T 24 (7.4)
Cladd H 26 (8.4)
Clark CJP 72 (P154)
Clark GWB 12 (1.3), 41 (P34)
Clark JE 35 (P9)
Clark JP 92 (P233)
Clark PI 44 (P46)
Clark T 31 (10.4)
Clarke I 25 (7.8)
Clarke NW 38 (P20), 38 (P21),
53 (P80)
Clarke PA 42 (P37), 55 (P84),
77 (P172)
Clayton JA 27 (8.7)
Coe S 42 (P39)
Cohen D 64 (P121)
Coleman N 108 (P294)
Coleman R 47 (P56)
Coleman RE 50 (P67)
Coles B 53 (P79)
Coletta PL 40 (P28)
Collard JG 9 (S28)
Collins HM 80 (P185), 83
(P195)
Collis SJ 38 (P20)
Colquitt J 44 (P47)
Colston KW 46 (P54), 47 (P55),
57 (P95)
Conn A 96 (P248)
Conway J 17 (3.7), 104 (P279)
Cook G 102 (P269)
Cook IH 33 (P4)
Cook M 42 (P36)
Cook S 88 (P216)
Coombes RC 45 (P48), 45 (P49)
Cooper CS 92 (P233)
Corfe B 32 (10.8)
Corke K 35 (P12)
Cornelius V 59 (P101)
Correa I 96 (P249)
Cosaert J 57 (P93), 95 (P243)
Coshic O 49 (P65), 101 (P265)
Cosset FL 24 (7.4)
Costello E 95 (P245)
Cottier B 17 (3.6)
Coulthard S 60 (P104), 60
(P105)
Court J 53 (P79)
Cowan R 38 (P20), 38 (P21)
Cowan RA 52 (P76), 61 (P108)
Coward D 27 (8.8)
Cowen R 14 (2.3)
Cowen RL 14 (2.2)
Cowie VJ 18 (3.8)
Craig AR 108 (P295)
Craven R 13 (1.8)
Crawford M 50 (P66)
Crawford SM 64 (P122)
CRC Phase I/II Clinical Trials
Committee 25 (7.7)
Crimmins J 87 (P211)
Crnograc-Jurcevic T 82 (P193)
Crolla JA 108 (P293)
Crosby T 21 (5.5), 43 (P43),
104 (P276), 104 (P277)
Crosland R 84 (P200)
Croucher P 47 (P56)
Crown J 20 (5.2)
Crowther D 61 (P108)
Cull A 18 (3.8), 66 (P128)
Cullen MH 64 (P120)
Cummings J 101 (P266)
Cunningham D 71 (P150)
Cunningham SH 24 (7.1), 24
(7.2)
Cure H 57 (P93)
Curry B 34 (P6), 34 (P7)
Curtin NJ 37 (P19), 81 (P188)
Curwen JO 34 (P7)
Cutress R 23 (6.7)
Cuzick J 2 (CT5)
Czajka A 25 (7.8)
Dachs GU 15 (2.5), 98 (P253)
Daley F 50 (P69)
Dalgleish A 25 (7.8)
Dallimore NS 68 (P138)
Dallman C 59 (P100)
Dallosso A 30 (10.1)
Daoud R 68 (P138)
D'Arcy S 62 (P112)
Das SN 101 (P265)
Davidson J 29 (9.7)
Davidson S 99 (P258)
Davies BR 56 (P91)
Davies CL 12 (1.3)
Davies G 86 (P208), 86 (P209)
Davies JS 27 (8.6)
Davies M 42 (P39)
Davison EV 30 (10.2)
Dawar R 49 (P65)
DCIS Working Party 2 (CT5)
de Bono JS 89 (P219), 94
(P238)
de Vries C 42 (P36)
Deakin DPD 61 (P108)
Dearling J 99 (P257)
Dearnaley D 15 (3.1), 27 (8.8)
Dearnaley DP 1 (CT4)
Delgado F-M 94 (P239)
Delord J-P 94 (P240)
Delves T 59 (P101)
Denic S 105 (P281), 105 (P282)
Denmeade SR 7 (S18)
Dent J 73 (P158)
Denton AS 68 (P135)
Dervan P 45 (P50)
Dervan PA 51 (P71)
Dewhurst O 83 (P196)
Diaz RM 24 (7.4)
Diaz-Mendez F 106 (P285)
Dickson J 89 (P220)
Dillon JF 37 (P18)
Dirix L 57 (P93)
Dive C 23 (6.5), 32 (10.8)
Dixon M 21 (5.6)
Djamgoz MBA 8 (S21)
Dobson PRM 100 (P262)
Dodson A 13 (1.6), 49 (P63), 81
(P189)
Dodwell D 50 (P66), 50 (P67)
Dogaru M 58 (P96)
Dojcinov S 72 (P151)
Dolan K 71 (P147)
Doran J 25 (7.7)
Dornan D 46 (P52)
Double JA 92 (P232)
Dovey GJ 13 (1.7)
Drake R 101 (P267)
Dredge K 25 (7.8)
D'Souza D 89 (P221)
Du Y 59 (P103)
Duddy P 79 (P181)
Duddy PM 82 (P194)
Dukes M 34 (P6), 34 (P7)
Dumaz N 5 (S11)
Duncan P 74 (P159)
Duncombe A 59 (P102)
Dunlop DJ 61 (P108)
Dunn J 61 (P111)
Dunn JA 50 (P67)
Durkacz BW 37 (P19), 80
(P183)
Durrant L 96 (P248)
Dutch ColoRectal Cancer Group
5 (S9)
Dutkowski CM 99 (P259), 99
(P260)
Dyer SA 30 (10.2)
Dyker K 29 (9.5)
Earl HM 50 (P67), 51 (P70)
Early A 71 (P148)
Easton D 21 (5.7)
Easty D 45 (P50), 51 (P71)
Eaton JD 97 (P251)
Eccles D 79 (P181)
Edelman M 100 (P264)
Eden OB 107 (P290), 107
(P291), 108 (P292)
Edinburgh Breast Group 28
(9.4)
Edmondson RJ 56 (P91)
Edwards J 74 (P159),
88 (P218)
Edwards S 92 (P233)
Eeles R 15 (3.1)
Eeles RA 21 (5.7)
Eisen T 82 (P191)
Elkak A 85 (P203)
Ell PJ 27 (8.5)
Elliiott M 106 (P286)
Ellis I 55 (P86)
Ellis P 20 (5.1), 20 (5.3)
Ellison D 39 (P25)
Ellison DW 30 (10.2), 108
(P293)
Elllis I 54 (P82)
Elsden JL 78 (P176)
Emett J 50 (P68)
Emmott J 90 (P226)
End DW 7 (S17)
English P 1 (CT3)
Erler J 23 (6.5)
Erridge SC 29 (9.7)
Errington J 93 (P235)
Essapen S 42 (P36)
Eswar CV 52 (P74)
Euden SA 40 (P30)
Evans MT 95 (P242)
Evans SA 81 (P190)
Evans T 20 (5.2)
Evans TRJ 75 (P165)
Everard JE 70 (P144)
Faluyi OO 40 (P28)
Farmery SM 78 (P178)
Farnie G 80 (P183), 81 (P188)
Farrugia D 88 (P217)
Fashola-Stone E 98 (P255)
Felix CA 31 (10.5)
Fenton JAL 75 (P163)
Ferguson CJ 62 (P114)
Fernando I 61 (P111)
Ferry DR 25 (7.7)
Field JK 58 (P97), 71 (P147)
Filbert FJ 9 (S27)
Fincham VJ 82 (P192)
Fingert H 89 (P219)
Fisher P 17 (3.7)
Fitzgibbon J 59 (P101)
Flanagan E 26 (8.1)
Fletcher-Monaghan AJ 85
(P205)
Flux G 102 (P269)
Flynn A 99 (P257)
Folkes LK 15 (2.5)
Foot N 31 (10.4)
Forbes H 17 (3.6)
Foresti R 35 (P9)
Forrest APM 21 (5.6)
Foskett M 26 (8.2)
Foster CS 13 (1.6), 49 (P63)
Frame MC 22 (6.2), 82 (P192)
Freemont P 109 (P296)
Fujiyama C 15 (2.7)
Fuller-Pace F 12 (1.4)
Fumoleau P 94 (P239), 94
(P241)
Furniss DLS 104 (P278)
Fyfe D 68 (P136)
Gabra H 73 (P155), 73 (P156),
73 (P157)
Gadd J 15 (3.1)
Galadari S 105 (P281)
Gambhir S 9 (S26)
Ganesan TS 26 (8.1)
Ganta S 87 (P211), 87
(P214)
Ganusevich I 91 (P227)
Garnett M 5 (S11)
Garrett MD 80 (P186)
Gastaldi T 91 (P228)
Gattamaneni HR 64 (P123)
Gaughan L 93 (P234), 100
(P261)
Gaze MN 18 (3.8)
Author index 111
Gazzard BG 61 (P109)
George RE 78 (P176)
George WD 2 (CT5)
Gerrard G 29 (9.5)
Gerrard GE 63 (P119)
Gerrard M 76 (P168)
Gerstain E 49 (P64)
Gescher A 40 (P29)
Gescher AJ 40 (P30), 40 (P31)
Ghaderi A 42 (P38), 48 (P62)
Ghaneh P 95 (P245)
Gharesi-Fard B 42 (P38), 48
(P62)
Ghilchick M 85 (P203)
Gibbs P 90 (P223)
Gilbertson RJ 108 (P293)
Gilchrist R 82 (P194)
Gilham DE 39 (P26), 97 (P250),
97 (P251), 97 (P252)
Gilhooley A 53 (P80)
Gill D 15 (2.6)
Gill J 75 (P166)
Gill JH 12 (1.1)
Gillespie D 18 (4.2)
Gillett C 20 (5.1)
Gilliam AD 55 (P84)
Gilligan D 62 (P115), 63 (P117)
Gilmore I 54 (P82), 55 (P86)
Gilmour J 43 (P40)
Gilmour LMR 56 (P89), 76
(P167)
Glaholm J 29 (9.6)
Glaholm JG 16 (3.3)
Glennie M 24 (7.3)
Glover C 43 (P41)
Glynne-Jones R 44 (P44)
Gnanapragasam VJ 88 (P216)
Godbout R 78 (P176)
Goddard JC 35 (P10)
Goddard PM 91 (P229)
Goepel JR 60 (P107)
Goetz A 94 (P238)
Goff LK 31 (10.4), 109 (P297)
Going JJ 74 (P159), 85 (P205)
Goldesbrough DR 72 (P154)
Golding BT 37 (P19), 81 (P188)
Goldspink G 42 (P39)
Goodchild K 48 (P61)
Gore DM 81 (P189)
Gore M 26 (8.1), 28 (9.2), 57
(P93)
Gosden RG 27 (8.7)
Goudie D 75 (P164)
Gower NH 95 (P242)
Goyal A 49 (P65), 101 (P265)
Graham RA 96 (P249)
Gray S 18 (4.3), 41 (P33)
Greaves M 11 (S33)
Greco O 15 (2.5), 98 (P253)
Green M 42 (P36)
Greenhalf W 54 (P82), 54
(P83), 55 (P85), 55 (P86)
Greening S 68 (P138)
Greenman J 90 (P225)
Gregor A 18 (3.8), 81 (P187)
Grieve R 20 (5.2)
Grieve RJ 65 (P126), 70 (P143)
Griffin RJ 37 (P19), 81 (P188)
Griffiths G 1 (CT3), 32 (10.8),
52 (P77)
Griffiths GO 52 (P76)
Griffiths L 98 (P254)
Grigor KM 52 (P76)
Grimer R 106 (P284)
Grimes S 77 (P172)
Grimsha MJ 19 (4.6)
Grimshaw D 83 (P198)
Grundy RG 30 (10.2)
Guest R 97 (P252)
Guillou PJ 12 (1.3), 41 (P34),
78 (P178), 83 (P196)
Gumbleton M 99 (P260)
Gupta NK 29 (9.6)
Gupta R 60 (P106)
Gupta RK 61 (P108)
Gupta S 42 (P39)
Gusterson BA 8 (S23)
Guthrie D 28 (9 .1)
Hackett P 64 (P121)
Hacking D 57 (P93)
Hadley J 81 (P187)
Hague A 101 (P268)
Hall A 60 (P104)
Hall AG 60 (P105)
Hall E 15 (3.1), 20 (5.3)
Hall J 85 (P206), 86 (P207)
Halloran CM 95 (P245)
Hamblin T 59 (P102)
Hamlin PJ 77 (P171)
Hanby AM 20 (5.1)
Hancock A 30 (10.1)
Hancock BW 28 (9.2), 70
(P144), 72 (P152), 72 (P153)
Hancock H 72 (P152), 72
(P153)
Hankin S 106 (P287)
Hankin SL 76 (P168)
Hansell D 75 (P165)
Hansen VN 54 (P81)
Hardcastle I 81 (P188)
Hardwick R 104 (P277)
Hare C 89 (P221)
Hargreave T 1 (CT3)
Harkin L 41 (P33)
Harkins L 18 (4.3)
Harmer C 69 (P140), 69 (P141)
Harmer CL 102 (P269)
Harnden P 13 (1.8)
Harndon P 88 (P215)
Harnet P 57 (P93)
Harper 26 (8.1)
Harper P 52 (P77)
Harrington KJ 24 (7.4)
Harris AL 4 (S6), 15 (2.7)
Harris M 27 (8.7)
Harrison G 28 (9.2)
Harrison M 71 (P149)
Harroway J 21 (5.7)
Hart I 107 (P289)
Hart IR 22 (6.1), 82 (P193), 82
(P194)
Hassan AB 39 (P27)
Hatton M 17 (3.7)
Hatton MQ 62 (P114), 104
(P278)
Hausner PF 100 (P264)
Havard T 104 (P277)
Hawkins RE 39 (P26), 97
(P250), 97 (P251), 97 (P252)
Hay TJ 101 (P267)
Hayes L 5 (S11)
Hayne D 89 (P221)
Hemingway DM 33 (P1), 33
(P2), 33 (P3)
Henderson D 31 (10.3)
Henderson DC 43 (P40)
Hendry J 63 (P118)
Hendry JH 19 (4.5), 37 (P17),
38 (P20), 38 (P21)
Heng Y 76 (P169)
Heng YM 76 (P170)
Hennequin LF 34 (P6), 34 (P7)
Henry AM 63 (P119)
Henwood J 40 (P28)
Herbert CW 55 (P85)
Herschman HR 9 (S26)
Hesslewood S 25 (7.7)
Hetherington J 1 (CT3)
Hickman J 32 (10.8)
Hidalgo M 94 (P238)
Higgins HB 57 (P92)
Highley M 52 (P77)
Hill KA 40 (P30)
Hill M 55 (P87)
Hill S 50 (P66)
Hill SA 48 (P61)
Hing S 31 (10.4)
Hirai K 93 (P236)
Hirst DG 14 (2.4)
Hiscox S 86 (P209)
Hislop RG 12 (1.4), 75 (P164)
Hoang TTV 78 (P175)
Hobbs S 100 (P263)
Hoctin-Boes G 95 (P243)
Hogarth L 60 (P104)
Holcombe C 30 (9.8), 48 (P59),
48 (P60)
Holwell SE 34 (P8)
Homer EG 18 (4.2)
Honeychurch J 24 (7.3), 59
(P103)
Hornigold N 12 (1.1), 75
(P166), 77 (P173)
Horsman J 72 (P152), 72 (P153)
Horwich A 15 (3.1), 27 (8.8)
Hoskin PJ 36 (P13), 44 (P44),
89 (P220), 90 (P226)
Hostomsky Z 37 (P19)
Houghton J 2 (CT5), 29 (9.6)
House A 65 (P127)
Howard GCW 18 (3.8)
Howard HC 50 (P67)
Howarth M 72 (P154)
Howell JA 39 (P27)
Howell WM 12 (1.2)
Howes N 54 (P82), 55 (P86)
Hoyes KP 19 (4.5)
Huang A 43 (P40), 43 (P41)
Hubbard A 90 (P225)
Huddart R 15 (3.1), 52 (P77)
Huddart RA 27 (8.8), 54 (P81)
Hughes C 97 (P252)
Hughes CM 14 (2.4)
Hulbert M 58 (P97)
Hull MA 40 (P28)
Hunter IA 78 (P178)
Hupp TR 37 (P18), 46 (P52), 79
(P182)
Hurley H 11 (S35)
Hussain SA 16 (3.3), 25 (7.7)
Hutcheon A 21 (5.4)
Hutcheson IR 99 (P259), 99
(P260)
Hutchinson I 103 (P272)
Hutchinson OC 89 (P222)
Hyde K 85 (P206)
ICON Collaborators 28 (9.1)
Illidge T 24 (7.3)
Illidge TM 59 (P103)
Ilyas M 39 (P25)
Innes HE 30 (9.8), 63 (P118)
Iqball S 98 (P254)
Ireson CR 40 (P30), 40 (P31)
Irlam JJ 39 (P26)
Ironside JW 81 (P187)
Irwin H 99 (P257)
Isaacs JT 7 (S18)
Ismail A 16 (3.2)
Jablons D 77 (P174)
Jack W 21 (5.6)
Jackman AL 92 (P233)
Jackson C 15 (3.1)
Jackson DP 101 (P267)
Jackson S 30 (10.1)
Jacobsen C 7 (S18)
Jagdev S 47 (P56)
James ND 16 (3.3), 17 (3.6)
James RD 61 (P108)
James RFL 33 (P1), 33 (P3)
Jarvis MC 48 (P59), 48 (P60)
Jassam N 41 (P33)
Jay G 27 (8.8)
Jayasundara SG 47 (P55)
Jayne D 83 (P196)
Jeffery K 66 (P130)
Jenkins BL 22 (6.3)
Jenkins P 85 (P203)
Jenkins TC 84 (P201), 84
(P202), 92 (P230)
Jiang W 86 (P208)
Jiang WG 83 (P197), 83 (P198),
84 (P199), 86 (P209)
Jodrell DI 101 (P266)
Joffe J 21 (5.5)
Joffe JK 50 (P66)
Johnson L 20 (5.3)
Johnson P 24 (7.3), 59 (P100),
59 (P102), 94 (P241)
Johnson PWM 59 (P103)
Johnson U 89 (P221)
Johnston D 13 (1.5)
Johnston DA 37 (P18)
Johnston S 21 (5.5)
Joiner M 15 (2.5), 36 (P16)
Joiner MC 16 (3.4)
Jolivet J 94 (P238)
Jones A 15 (2.7), 20 (5.2)
Jones D 40 (P31)
Jones DJL 40 (P30)
Jones JL 35 (P10)
Jones K 17 (3.6)
Jones LK 31 (10.4), 107 (P288)
Jones RD 29 (9.7), 104 (P278)
Jordan SD 57 (P92)
Joseph J 63 (P119)
Jouanneau J 10 (S30)
Jowsey PA 80 (P183)
112 Author index
Jubb A 18 (4.3)
Judson I 94 (P241)
Julyan PJ 25 (7.7)
Kan O 98 (P254)
Kang Y 55 (P86)
Kanthou C 35 (P9)
Kapiteijn E 5 (S9)
Karapetis C 26(8.1)
Kay G 92 (P232)
Kaya G 92 (P231)
Kaye S 25 (7.5)
Kaye SB 5 (S12)
Kazemi Noureini S 85 (P204)
Keilty G 14 (2.4)
Keith WN 24 (7.1), 85 (P205)
Kelland LR 100 (P263)
Kelly CG 18 (3.8)
Kelsey AM 107 (P290), 107
(P291)
Kendrew J 34 (P6), 34 (P7)
Kennedy I 20 (5.2)
Kennedy W 77 (P173), 79
(P180)
Kenyon RM 78 (P176)
Kerby I 72 (P151)
Kernohan NM 12 (1.4), 13
(1.5), 46 (P52)
Kerr DJ 25 (7.7)
Kerr G 21 (5.6), 28 (9.4)
Kerr GR 18 (3.8)
Keshtgar MRS 27 (8.5)
Khan SR 7 (S18)
Khanduri S 65 (P126)
Khanna S 57 (P94)
Khelifi AF 35 (P9)
Kiely M 65 (P127), 66 (P128)
Kiltie AE 38 (P22)
Kim J 37 (P17)
Kingsman S 98 (P254)
Kirillova N 97 (P252)
Kirkpatrick KL 85 (P203)
Kirn D 25 (7.5)
Kitching M 81 (P188)
Knowlden JM 99 (P259)
Knowles M 79 (P180)
Knowles MA 12 (1.1), 74
(P160), 74 (P161), 74 (P162),
75 (P166), 77 (P173), 80
(P184)
Kockelbergh RC 35 (P10)
Koehler M 95 (P243)
Koldaeva E 49 (P64)
Komolmit P 77 (P171)
Kothari MS 45 (P48), 45 (P49)
Krishna NS 88 (P218)
Kuhn J 89 (P219)
Kumar A 49 (P65)
Kumar P 107 (P289)
Kumar S 107 (P289)
Kunkler IH 21 (5.6)
Kushlinsky N 49 (P64)
Kynaston H 53 (P79)
Kynaston HG 27 (8 .6)
Laban C 85 (P203)
Ladanyi M 11 (S34)
Lahiri M 47 (P57)
Laing R 16 (3.2)
Lakhani S 27 (8.5)
Lane J 83 (P198)
Lang SH 85 (P206)
Langdon SP 56 (P88), 56 (P89),
76 (P167)
Langley S 16 (3.2)
Last KW 59 (P101)
Laterra J 83 (P198)
Laughton CA 103 (P272)
Lawrence D 15 (3.1), 20 (5.3)
Lawrie LC 43 (P42)
Lawry 60 (P107)
Lawry J 76 (P168), 106 (P287)
Layton C 51 (P72)
Lechler R 26 (8.1)
Lee A 88 (P217)
Lee AC 27 (8.5)
Leer JWH 5 (S9)
Lehane M 97 (P252)
Lemoine N 26 (8.1)
Leonard R 26 (8.3)
Leonard RCF 20 (5.2), 21 (5.4),
21 (5.6), 28 (9.4), 50 (P67), 67
(P134)
Leong CO 103 (P272)
Leslie M 60 (P105)
Lester J 72 (P151)
Leung HY 22 (6.3), 86 (P210),
87 (P213)
Levay J 20 (5.2)
Levitin I 91 (P227)
Levy D 29 (9.5)
Lewis CE 15 (2.6), 35 (P12), 36
(P15)
Lewis IJ 31 (10.5), 106 (P284)
Lewis W 104 (P277)
Li AGK 12 (1.3), 41 (P34)
Li L 73 (P155)
Lian L-Y 14 (2.2)
Liang Q 9 (S26)
Lieberman BA 27 (8.7)
Light Y 5 (S11)
Lillington D 31 (10.4)
Liloglou T 58 (P97)
Lim CK 40 (P31)
Linch D 72 (P152)
Linch DC 2 (CT7)
Lind M 90 (P225)
Lindahl T 38 (P22)
Linder B 107 (P288)
Lister TA 59 (P101), 60 (P106),
61 (P108)
Little M 60 (P104)
Liu D 89 (P222)
Livingstone A 24 (7.1)
Livni N 45 (P48)
Loadman PM 87 (P211)
Loane JF 41 (P35)
Lockyer SL 36 (P14)
Lofts F 55 (P87)
Lombard M 54 (P82)
Lombard MG 55 (P86)
Loncaster J 35 (P11)
London NJM 33 (P1), 33 (P2),
33 (P3)
Lorigan P 51 (P72)
Louhelainen J 75 (P166)
Louhelainen JR 74 (P160)
Low SE 72 (P152)
Lowe J 50 (P68), 90 (P226)
Lowry M 90 (P224)
Lucie NP 61 (P108)
Lunec J 53 (P78), 78 (P176), 78
(P177), 80 (P183), 81 (P188)
McAdam K 51 (P70)
McAndrew C 80 (P186)
McArdle C 1 (CT2)
McArdle L 45 (P50), 51 (P71)
Macbeth F 63 (P116)
McCabe HL 71 (P148)
McCarragher LSM 100 (P262)
McCarthy H 59 (P102)
McCarthy HO 14 (2.4)
McClelland H 66 (P130)
McCluskey 24 (7.1)
McCormack M 62 (P113), 68
(P137)
Macdonald CD 47 (P55), 57
(P95)
McDonald FE 81 (P187)
MacDougall RH 18 (3.8), 29
(9.6)
McDowell HP 106 (P286)
McFadyen MCE 19 (4.7)
Machesky L 22 (6.2)
McIllmurray M 21 (5.5)
McIntyre G 20 (5.2)
McJury M 17 (3.7)
McKay JA 41 (P35)
Mackay SP 84 (P201)
McKeown SR 14 (2.4), 52 (P75)
McKerrecher D 34 (P6)
MacKie R 107 (P289)
MacLaren D 9 (S26)
McLelland HR 40 (P30)
McLeod HL 41 (P35)
Macleod K 56 (P88)
Macleod KG 76 (P167)
McMahon G 33 (P2)
McMurray A 52 (P74)
McNally RJQ 107 (P290), 107
(P291)
MacNeil S 51 (P72)
McNeish IA 26 (8.2)
MacPartlin M 18 (4.2)
McWilliams DF 42 (P37), 55
(P84), 77 (P172), 81 (P190)
Madhusudan S 26 (8.1)
Magee CJ 54 (P83)
Magee L 62 (P115), 63 (P117)
Maher EJ 68 (P135)
Mahmoudi M 56 (P90)
Mairs RJ 24 (7.1), 24 (7.2)
Maisey NR 71 (P150)
Maitland NJ 7 (S20), 23 (6.6),
85 (P206), 86 (P207), 87
(P212)
Makin G 32 (10.8)
Makin WP 65 (P125)
Makris A 26 (8.4), 44 (P44), 48
(P61), 50 (P68), 50 (P69), 90
(P226)
Makris M 26 (8.4)
Malekhosseini A 42 (P38)
Malik KTA 30 (10.1)
Maloney P 58 (P97)
Mangham C 106 (P284)
Mansel RE 83 (P198), 84
(P199), 86 (P209)
Mansi J 20 (5.2), 50 (P67)
Mansi JL 47 (P55), 57 (P95)
Manson J 104 (P277)
Manson MM 40 (P30)
Manton DJ 90 (P225)
Marais R 5 (S11)
Maravcyas A 55 (P87), 71
(P148), 90 (P225)
Margison GP 38 (P20), 38 (P21)
Marijnen CAM 5 (S9)
Markham A 73 (P158)
Markham AF 77 (P171)
Marks C 42 (P36)
Marland DM 101 (P268)
Marples B 15 (2.5), 36 (P16)
Marples M 51 (P73), 56 (P90)
Marriott J 25 (7.8)
Marshall E 44 (P46), 52 (P74),
63 (P118)
Marshall J 69 (P140)
Marshall JF 22 (6.1), 82 (P194)
Martin A 47 (P57)
Martin C 58 (P98)
Martin J 47 (P57), 60 (P107)
Martin L 48 (P59)
Martin N 37 (P19)
Martin TA 83 (P198), 84 (P199)
Marty M 94 (P239)
Mason AB 54 (P83)
Mason M 86 (P208)
Mason MD 27 (8.6), 53 (P79),
69 (P139)
Massey C 73 (P157)
Massie C 28 (9.4)
Mather SJ 39 (P25)
Matsumoto K 86 (P209)
Matthews S 68 (P135)
Maughan T 1 (CT1), 69 (P139),
72 (P151), 104 (P276)
Maughan TS 64 (P121)
Mayer A 65 (P124)
Mayles H 61 (P110)
Meczes EL 102 (P270)
Mehta PB 22 (6.3), 87 (P213)
Meijer L 6 (S15)
Melcher A 103 (P275)
Melcher L 62 (P113), 68 (P137)
Mellon JK 53 (P78)
Melvin WT 19 (4.7)
Meredith DM 108 (P295)
Meyer T 51 (P73), 56 (P90)
Michael A 55 (P87), 57 (P95)
Michael NP 98 (P255)
Mikhaeel NG 62 (P112), 69
(P142)
Miles AE 46 (P54)
Miles DW 25 (7.6), 96 (P249)
Miller EP 73 (P155), 73 (P156)
Mills JA 70 (P143)
Milroy R 99 (P258)
Mincher DJ 92 (P232)
Minchera DJ 92 (P231)
Minto L 60 (P104)
Minton N 98 (P256)
Minton NP 98 (P255)
Mishra MC 101 (P265)
Mitchell G 21 (5.7)
Mitra SS 43 (P43)
Modi A 90 (P225)
Modjtahedi H 42 (P36), 42
(P38), 48 (P62)
Author index 113
Moffitt DD 16 (3.3)
Mohith A 61 (P109)
Mokbel K 85 (P203)
Molife R 51 (P72)
Monaghan JM 56 (P91)
Monson KM 29 (9.6)
Moody M 65 (P124)
Moore JS 46 (P54)
Moore JV 53 (P80)
Moorman A 13 (1.7)
Moorwood K 30 (10.1)
Morgan B 57 (P94)
Morgan GJ 13 (1.7), 75 (P163)
Morgan MR 22 (6.1)
Morley SE 25 (7.5)
Morley-Jacob CA 108 (P294)
Morris R 101 (P266)
Morris TM 55 (P84)
Morrison V 64 (P121)
Mort D 104 (P276)
Motterlini R 35 (P9)
Moynihan C 27 (8.8)
MRC Bladder Cancer Group 1
(CT3), 52 (P76)
MRC Bladder Cancer Working
Party 52 (P77)
MRC Colorectal Cancer Group
1 (CT1), 28 (9.3), 44 (P45)
MRC and EORTC Colorectal
Cancer Groups 1 (CT2)
MRC PR05 Collaborators 1
(CT4)
Muers M 64 (P120)
Mukherjee R 88 (P218)
Mukherjee S 66 (P131)
Mullen P 76 (P167)
Muller G 25 (7.8)
Munro AJ 37 (P18)
Murdoch H 98 (P255)
Murdoch T 52 (P74)
Murphy PM 92 (P230)
Murray GI 19 (4.7), 41 (P35),
43 (P42)
Murray JC 76 (P169), 76 (P170)
Murray M 14 (2.4)
Murray PV 70 (P146)
Nacheva E 108 (P294)
Nagtegaal ID 5 (S9)
Nakamura T 86 (P209)
Nanus DM 87 (P212)
Napier M 51 (P73)
Nash GE 34 (P5)
Natrajan R 74 (P160)
Naylor CS 97 (P250)
Naylor S 98 (P254)
Neal DE 22 (6.3), 86 (P210), 87
(P213), 88 (P216), 93 (P234),
100 (P261)
Neat M 107 (P288)
Neave F 21 (5.5)
Nelson B 106 (P286)
Nelson M 61 (P109)
Nelstrop AE 56 (P90)
Neoptolemos JP 54 (P82), 55
(P86), 95 (P245)
Nevin GB 52 (P75)
Newcombe RG 68 (P138)
Newell DR 37 (P19), 81 (P188)
Newlands ES 26 (8.2)
Ng A 108 (P292)
Ng FL 46 (P54)
Nicholls J 27 (8.8)
Nicholson JC 108 (P293)
Nicholson KM 45 (P51)
Nicholson RI 99 (P259), 99
(P260)
Nishiyama H 12 (1.1)
Noble S 50 (P69)
Noon E 66 (P129)
Norman A 27 (8.8), 71 (P150)
Northen J 81 (P188)
Norton A 60 (P106), 70 (P146)
Nouri AME 96 (P246)
Nussey F 26 (8.3)
Nutt JE 53 (P78)
Nystrom M 103 (P274)
Nystrom ML 95 (P242)
O'Brien C 72 (P151)
O'Brien MER 70 (P146)
O'Brien S 103 (P272)
O'Byrne K 33 (P2), 57 (P94)
O'Byrne KJ 35 (P10)
Ochoa L 89 (P219)
O'Connor D 54 (P83)
O'Donnell A 94 (P241)
O'Donnell AJM 56 (P88)
O'Flynn K 53 (P80)
Ogilvie DJ 34 (P6), 34 (P7)
Oglesby SD 37 (P18)
Oliver RTD 88 (P217), 96
(P246), 96 (P247), 103 (P273),
103 (P274)
Olsburgh J 95 (P244)
O'Mahoney S 64 (P121)
O'Neil A 97 (P251), 97 (P252)
O'Neill LP 19 (4.4)
O'Neill P 13 (1.6), 49 (P63)
O'Neill PA 44 (P46)
Ongunkolade W 85 (P203)
O'Reilly S 21 (5.5)
O'Reilly SM 30 (9.8), 44 (P46)
O'Riordain DS 78 (P178)
Orr S 40 (P31)
Osborne M 44 (P44), 90 (P226)
Osinsky S 91 (P227)
Osman A 57 (P94)
Osman S 89 (P222)
Ostler P 89 (P220)
Ottensmeier C 59 (P102), 94
(P241)
Ottley CJ 102 (P271)
Packham G 23 (6.7), 46 (P53),
59 (P100)
Palmer DH 25 (7.7)
Pappin DJ 13 (1.8)
Pardo OE 22 (6.4)
Parker C 107 (P289)
Parker CA 36 (P15)
Parker D 72 (P154)
Parkins CS 34 (P5)
Parkins PCS 33 (P4)
Parmar M 1 (CT3), 27 (8.6)
Parr AM 64 (P123), 65 (P125)
Parr C 83 (P197), 86 (P209)
Patel D 26 (8.2)
Patel H 43 (P41)
Patel K 88 (P215)
Patel N 31 (10.4), 109 (P297)
Patel PM 101 (P267)
Patnaik A 89 (P219)
Patterson A 14 (2.3)
Patterson AV 14 (2.2)
Payne H 89 (P221)
Payne HA 62 (P113), 68 (P137)
Peace J 64 (P122)
Peake D 16 (3.3)
Pears R 21 (5.7)
Pearson ADJ 78 (P176), 78
(P177), 93 (P235), 102 (P270)
Pearson DG 102 (P271)
Pedley RB 98 (P256), 99 (P257)
Pemberton GE 57 (P92)
Perkins S 40 (P29)
Perren T 20 (5.2), 21 (5.5), 67
(P133), 73 (P158)
Perren TJ 50 (P66)
Perry PJ 84 (P202)
Perry RH 108 (P293)
Peto J 29 (9.6)
Pettersson S 92 (P232), 92
(P231)
Phelps M 9 (S26)
Pickard CDO 27 (8.5)
Pietsch T 79 (P179)
Pignatelli M 26 (8.1)
Pinel M-C 94 (P240), 94 (P241)
Pirmohamed M 40 (P30)
Pitsiouni M 109 (P297)
Pitt E 12 (1.1), 75 (P166)
Pittam M 50 (P68), 50 (P69)
Ple P 34 (P6)
Pledge S 17 (3.7)
Plumb J 18 (4.1), 99 (P258)
Plummer SM 40 (P30)
Plunkett TA 25 (7.6), 96 (P249)
Poole CJ 50 (P67), 57 (P92)
Porteous DJ 73 (P156), 73
(P157)
Porter KE 33 (P1), 33 (P2), 33
(P3)
Poston G 44 (P47)
Potter BVL 46 (P53)
Potter V 96 (P248)
Potts H 81 (P187)
Poulsom R 20 (5.1), 39 (P25)
Povey S 20 (5.2), 67 (P134)
Powell M 60 (P106)
Powell MEB 17 (3.5)
Powell MG 44 (P44)
Power DA 68 (P137), 69 (P142)
Powles T 61 (P109)
Poyner R 25 (7.7)
Poynton C 72 (P151)
Pratt N 75 (P164)
Pratt NR 13 (1.5)
Prebble EJ 30 (10.2)
Price A 18 (3.8), 29 (9.7)
Price C 21 (5.5)
Price P 64 (P121), 89 (P222)
Price PM 9 (S25)
Price S 66 (P129)
Primrose J 44 (P47)
Pring CM 41 (P34)
Prise VE 35 (P9)
Pritchard-Jones K 31 (10.4)
Protheroe AS 50 (P66)
Prouix L 94 (P238)
Przkora R 79 (P179)
Pulimood AB 87 (P213)
Puozzo C 94 (P241)
Purohit A 46 (P53)
Quigg M 24 (7.1)
Quinn MJ 68 (P137)
Quirke P 18 (4.3), 41 (P33)
Rabiasz GJ 73 (P156), 73
(P157)
Radford JA 27 (8.7),
61 (P108)
Radstone C 72 (P153)
Radvanyi F 10 (S30)
Rafferty M 45 (P50), 51 (P71)
Raguz S 22 (6.4)
Rahilly M 75 (P165)
Rainey DR 108 (P295)
Ramage J 96 (P248)
Ramani VAC 38 (P21)
Ramsay A 31 (10.3)
Ravanat JL 38 (P22)
Raynaud FL 91 (P229)
Raz A 10 (S31)
Redfern C 60 (P104)
Redfern CPF 93 (P235)
Reed MJ 46 (P53)
Rees M 44 (P47)
Reid R 81 (P188)
Renshaw M 66 (P129)
Rice K 76 (P169)
Richards E 61 (P110)
Richman PI 50 (P69)
Richmond GHP 34 (P7)
Richmond I 73 (P158)
Rigoreau L 37 (P19)
Rischin D 57 (P93)
Ritchie AA 101 (P266)
Ritchie JL 52 (P75)
Robbe I 64 (P121)
Robbins A 76 (P169)
Roberts I 108 (P294)
Roberts P 31 (10.5)
Roberts S 35 (P11)
Roberts SA 19 (4.5), 38 (P20),
38 (P21)
Robertson A 75 (P164)
Robertson G 17 (3.6)
Robins RA 81 (P190)
Robinson G 38 (P23)
Robinson H 18 (4.2)
Robinson MH 17 (3.7), 104
(P279)
Robinson PA 41 (P34), 77
(P171), 78 (P178)
Robinson S 14 (2.3)
Robinson SK 14 (2.2)
Robson CN 22 (6.3), 87 (P213),
88 (P216), 93 (P234), 100
(P261)
Robson T 14 (2.4)
Rodger A 21 (5.6)
Rodwell S 50 (P66)
Rogers C 68 (P138)
Rogers S 62 (P114)
Rogers TK 62 (P114)
Rohatiner AZS 59 (P101), 60
(P106), 61 (P108)
Rollinson S 13 (1.7)
114 Author index
Roman E 13 (1.7)
Rosoiu N 58 (P96)
Rosolen A 91 (P228), 105
(P280)
Ross EL 39 (P25)
Ross FM 108 (P293)
Ross VG 41 (P35)
Rossi A 61 (P108)
Rostami-HA 47 (P56)
Rothman MT 17 (3.5)
Rowan A 20 (5.1)
Rowinsky EK 89 (P219), 94
(P238)
Roy AEF 71 (P149)
Royds JA 36 (P15)
Ruchatz A 24 (7.4)
Rudd RM 95 (P242)
Russell JM 52 (P76)
Russell SA 27 (8.7)
Russell SG 38 (P23), 62 (P115),
63 (P117)
Russell SJ 24 (7.4)
Russell ST 93 (P236)
Rustin GJS 51 (P73), 56 (P90)
Rutherford J 79 (P181)
Rutten HJT 5 (S9)
Ryder WDJ 61 (P108)
Rye R 81 (P187)
Saad Aldeen JA 60 (P107)
Sadler PJ 101 (P266)
Sadozye AH 70 (P145)
Saffie R 96 (P246), 96 (P247)
Saha V 31 (10.4), 107 (P288),
109 (P296), 109 (P297)
Salazar R 21 (5.4)
Salerno G 22 (6.4)
Sales M 75 (P164)
Samuel D 109 (P297)
Samuel K 26 (8.3)
Sanger VK 38 (P20), 38 (P21)
Sarela A 12 (1.3)
Sartori F 105 (P280)
Satyamurthy N 9 (S26)
Saunders MI 16 (3.4)
Sawyer EJ 20 (5.1)
Sawyer W 29 (9.6)
Scholefield JH 83 (P195)
Schulz WA 23 (6.8)
Schwartz G 89 (P219)
Scott D 73 (P155)
Scott DK 78 (P177)
Scott S 36 (P16)
Scott SD 15 (2.5)
Scottish Cancer Therapy
Network 29 (9.7)
Scottish Lung Cancer Trails
Group 29 (9.7)
Seargent J 87 (P214)
Seaton A 78 (P175)
Sebag-Montefiore D 28 (9.3)
103 (P275)
Seckl M 26 (8.1)
Seckl MJ 22 (6.4),
26 (8.2)
SECRAB Steering Committee
61 (P111)
Seifert HH 23 (6.8)
Selby P 8 (S24), 65 (P127), 67
(P133), 95 (P244)
Selby PJ 13 (1.8), 66 (P128), 67
(P132), 88 (P215), 101 (P267)
Sellar GC 73 (P155), 73 (P156),
73 (P157)
Sewell JM 56 (P89)
Seymour LW 25 (7.7)
Seymour M 44 (P45)
Shaaban A 13 (1.6), 49 (P63)
Shackley DC 53 (P80)
Shah N 16 (3.4), 44 (P44)
Shahidi M 27 (8.8)
Shalet SM 27 (8.7)
Shamash J 60 (P106), 61
(P108), 95 (P242), 103 (P274)
Sharma RA 40 (P29), 40 (P30)
Sharma SK 98 (P256), 99
(P257)
Sharrard RM 23 (6.6)
Shawcross SG 107 (P289)
Sheddon M 52 (P74)
Sheer D 109 (P296)
Shefford KE 101 (P268)
Shelley MD 53 (P79)
Sheppard F 14 (2.2)
Shing DC 108 (P294)
Shoemaker RH 6 (S14)
Shoker BS 48 (P59), 48 (P60)
Short SC 102 (P269)
Shotton C 42 (P36)
Shousha S 45 (P48), 45 (P49)
Shubina I 49 (P64)
Shulkes A 41 (P32)
Sibley K 75 (P163), 79 (P180)
Sibtain A 36 (P13), 48 (P61)
Sigan A 91 (P227)
Simcock R 66 (P129)
Simmonds P 44 (P47)
Simmons C 94 (P238)
Sims EC 17 (3.5)
Sims MA 98 (P255)
Singh R 40 (P29)
Sinnett HD 45 (P48)
Sisley K 107 (P289)
Skelton LA 92 (P233)
Skliris G 41 (P33)
Slade M 45 (P48)
Slade MJ 45 (P49)
Sloane JP 48 (P59), 48 (P60)
Smalley KSM 82 (P191)
Smart H 54 (P82)
Smart HL 55 (P86)
Smirnova G 49 (P64)
Smith A 65 (P127), 67 (P133)
Smith AB 67 (P132)
Smith DB 44 (P46)
Smith IE 70 (P146)
Smith JC 27 (8.6)
Smith JM 26 (8.4), 101 (P267)
Smith KL 19 (4.4)
Smith M 95 (P243)
Smith NF 91 (P229)
Smith P 2 (CT7), 72 (P152)
Smith V 100 (P263)
Smyth JF 56 (P88), 56 (P89), 73
(P155), 73 (P156), 73 (P157),
76 (P167)
Snary D 39 (P25)
Sorensen N 79 (P179)
Sothi S 65 (P126)
Soutar DS 25 (7.5)
Southgate J 95 (P244)
Speake WJ 80 (P185)
Spearman H 98 (P254)
Speight PM 22 (6.1)
Speirs V 41 (P33)
Spencer DIR 98 (P256)
Spendlove I 96 (P248)
Spiller D 81 (P189)
Spittle MF 69 (P142)
Srivastava A 49 (P65), 101
(P265)
Stafford H 82 (P194)
Staffurth JN 54 (P81)
Stahlschmidt J 18 (4.3)
Stanley LA 78 (P175)
Stark D 65 (P127), 67 (P133)
Stark M 85 (P206)
Staub J 39 (P25)
Steel CM 75 (P164)
Steele JPC 103 (P273), 103
(P274)
Steele PCJ 95 (P242)
Steinhoff C 23 (6.8)
Stephens TC 100 (P264)
Stephenson Jnr J 94 (P238)
Steup WH 5 (S9)
Stevens AM 70 (P143)
Stevens MFG 103 (P272)
Stevenson F 59 (P102)
Steward J 68 (P138)
Steward WP 40 (P29), 40 (P30),
40 (P31)
Stewart M 73 (P155), 81 (P187)
Stirling D 25 (7.8)
Stockley M 37 (P19)
Stocks SC 13 (1.5), 75 (P164)
Stockton P 63 (P118)
Stockwell SR 80 (P186)
Stokes ESE 34 (P6), 34 (P7)
Stower M 1 (CT3), 85 (P206),
86 (P207)
Stratford I 14 (2.3), 23 (6.5), 35
(P11)
Stratford IJ 14 (2.2), 36 (P14),
39 (P24)
Strathdee G 18 (4.1),
99 (P258)
Strauss SJ 51 (P73)
Streuli CH 45 (P51)
Strickland S 26 (8.2)
Stronach E 73 (P156)
Stronach EA 73 (P157)
Stuart N 21 (5.5), 69 (P139)
Stuart R 85 (P205), 94 (P240)
Sun RJ 100 (P264)
Sutton CD 35 (P10)
Sutton R 55 (P86), 71 (P147),
81 (P189)
Swiatkowski S 23 (6.8)
Sydes MP 1 (CT4)
Symonds RP 57 (P94)
Syndikus I 61 (P110)
Talei A 48 (P62)
Tannock IF 3 (S2), 20 (4.8)
Taylor GM 108 (P292)
Taylor GR 77 (P171)
Taylor-Papadimitriou J 25 (7.6),
96 (P249)
Tchemeris G 49 (P64)
Telfer BA 14 (2.2), 36 (P14), 39
(P24)
Teo NB 48 (P59), 48 (P60)
Tham BWL 70 (P144)
Thavaraj SJ 101 (P268)
Theaker JM 12 (1.2)
Thiery JP 10 (S30)
Thilak L 87 (P213)
Thistlethwaite A 32 (10.8)
Thomas AL 57 (P94), 66 (P130)
Thomas GJ 22 (6.1)
Thomas H 26 (8.1), 42 (P36)
Thomas NPB 99 (P260)
Thompson AM 13 (1.5), 37
(P18), 46 (P52), 75 (P164), 79
(P182)
Thompson I 89 (P219)
Thompson MJ 87 (P211), 87
(P214)
Thomson CS 29 (9.7)
Thorp N 61 (P110)
Thorp NC 70 (P143)
Thorpe P 45 (P48)
Threadgold J 54 (P82)
Tilby MJ 102 (P270), 102
(P271)
Timpson P 22 (6.2)
Tisdale MJ 93 (P236), 93 (P237)
Tobias JS 29 (9.6)
Tocher AW 89 (P219)
Todd I 76 (P169)
Tolhurst RS 45 (P49)
Tollenaar RAEM 5 (S9)
Tombal B 7 (S18)
Tomlinson IPM 20 (5.1)
Tonn JC 79 (P179)
Topham C 42 (P36)
Torabi-Pour N 96 (P246), Pour
N 96 (P247)
Totty N 13 (1.8)
Townsend PA 23 (6.7)
Toy E 20 (5.2), 63 (P116), 72
(P151), 104 (P276), 104
(P277)
Tozer D 90 (P225)
Tozer DJ 90 (P223)
Tozer GM 15 (2.5), 33 (P4), 34
(P5), 35 (P9), 98 (P253)
Trapani V 103 (P272)
Tsavellas G 43 (P41)
Tupper J 98 (P253)
Turnbull A 92 (P231), 92 (P232)
Turnbull LW 90 (P223), 90
(P224), 90 (P225)
Turner AJ 87 (P212)
Turner BM 19 (4.4)
Turner LA 28 (9.2)
Turner M 26 (8.3)
Turner SJ 12 (1.2)
Twelves C 21 (5.4), 50 (P67)
Ubhi BTS 108 (P295)
UK Capecitabine Audit Group
21 (5.4)
UKHAN Trial Group 29 (9.6)
Underwood MA 88 (P218)
Unwin RD 13 (1.8)
Uscinska B 52 (P77)
Uscinska BM 52 (P76)
Usmani BA 87 (P212)
Author index 115
116 Author index
Valentine A 52 (P75)
van de Velde CJH 5 (S9)
van den Brink M 5 (S9)
van Krieken JHJM 5 (S9)
Variend S 76 (P168)
Variol P 94 (P240)
Vasei M 42 (P38), 48 (P62)
Vashisht R 45 (P48)
Vaughan T 98 (P255)
Veal GJ 93 (P235)
Velikova G 67 (P132), 67
(P133)
Verbecke C 41 (P34)
Verbeke C 12 (1.3)
Vermorken J-B 94 (P240)
Verrill M 50 (P67)
Verschoyle RD 40 (P31)
Veuger S 37 (P19)
Vickery B 67 (P134)
Vilarino-Varela M 62 (P113)
Vile RG 24 (7.4)
Vini L 69 (P140), 69 (P141),
102 (P269)
Vlatkovic N 81 (P189)
Von Hoff DD 94 (P238)
Vrieling H 38 (P22)
Wadsworth PF 34 (P7)
Waite K 62 (P115)
Waite KJ 63 (P117)
Walker DA 108 (P293)
Walker SJ 71 (P147)
Wallace D 1 (CT3)
Wallace DMA 16 (3.3)
Walshaw MJ 63 (P118)
Wang B 90 (P224)
Warbrick E 37 (P18)
Ward M 65 (P124)
Ward W 76 (P169)
Wardman P 15 (2.5)
Warner TD 17 (3.5)
Warren J 61 (P110)
Warrington A 15 (3.1)
Wasan H 55 (P87)
Waterfall M 26 (8.3)
Waterton JC 34 (P7)
Watson AJ 27 (8.7)
Watson M 71 (P150)
Watson SA 42 (P37), 55 (P84),
77 (P172), 80 (P185), 81
(P190), 83 (P195)
Watt KP 73 (P155)
Watters AD 74 (P159), 88
(P218)
Webber SE 37 (P19)
Wedge SR 34 (P6), 34 (P7), 39
(P24)
Weeraratna A 7 (S18)
Weitman S 89 (P219)
Wells M 15 (2.6), 35 (P12), 84
(P200)
Welsh Cancer Care for the
Elderly Research Group 64
(P121)
Welsh P 24 (7.2)
West AF 86 (P210)
West C 3 (S4), 35 (P11)
West CML 37 (P17)
Weston P 1 (CT3)
Westwell AD 103 (P272)
Westwood G 32 (10.7)
Wheatley DA 69 (P141)
Wheatley K 28 (9.2)
Wheelhouse RT 92 (P230)
Whelan P 1 (CT3), 88 (P215)
White M 81 (P189)
White MRH 106 (P286)
Whitehouse AS 93 (P237)
Whitehurst C 53 (P80)
Wiestler OD 79 (P179)
Wiggers T 5 (S9)
Wijnhoven SW 38 (P22)
Wild CP 13 (1.7)
Wilkes SJ 109 (P297)
Wilkinson N 73 (P158)
Wilkinson PM 61 (P108)
Wilkinson RW 39 (P25)
Wilks DP 37 (P17)
Willett EV 13 (1.7)
Williams G 104 (P277)
Williams GT 68 (P138)
Williams K 14 (2.3),
23 (6.5)
Williams KJ 14 (2.2), 36 (P14),
39 (P24)
Williams ML 40 (P30),
40 (P31)
Williams SV 74 (P161), 74
(P162)
Wilson A 59 (P101),
60 (P106)
Wilson CB 51 (P70)
Wilson DC 70 (P143)
Wilson GD 36 (P13),
50 (P69)
Wilson L 24 (7.1)
Wilson P 103 (P274)
Wilt T 53 (P79)
Windmill E 25 (7.6)
Winslet M 42 (P39)
Withey J 31 (10.6)
Wong T 55 (P86)
Wong WL 50 (P68),
90 (P226)
Woo LW 46 (P53)
Wood L 46 (P53)
Workman P 4 (S5),
89 (P222), 91 (P229), 100
(P263)
Wright EP 66 (P128)
Wright P 67 (P133)
Wu PJ 107 (P288),
109 (P296)
Wyatt E 81 (P187)
Wyllie AH 5 (S10)
Xinarianos G 58 (P97)
Yafai F 92 (P233)
Yagui-Beltran A 77 (P174), 79
(P182)
Yahaya H 45 (P49)
Yazdanbod M 85 (P204)
Yip D 26 (8.1)
Yorkshire Breast Cancer
Research Group 50 (P66)
You L 77 (P174)
Young AM 25 (7.7)
Young BD 107 (P288)
Zalutsky MR 24 (7.2)
Zhu G 82 (P193)
Ziaee AA 85 (P204)
Ziyaie D 46 (P52)

				

